# Differences in Preventive Care Uptake in Attached and Unattached Rural-Living Residents

**DOI:** 10.1177/15248399251350623

**Published:** 2025-06-28

**Authors:** Lindsay Burton, Kathy L. Rush, Cherisse L. Seaton, Mindy A. Smith, Kendra Corman, Eric P. H. Li

**Affiliations:** 1 The University of British Columbia-Okanagan, Kelowna, British Columbia, Canada; 2 Patient Voices Network, Vancouver, British Columbia, Canada; 3 Michigan State University, East Lansing, MI, USA

**Keywords:** prevention, unattached patients, access to care, health promotion, screening, rural

## Abstract

Preventive care services are crucial for overall health, yet, rural communities experience low rates of preventive service use. Primary care providers are pivotal in facilitating preventive service uptake (e.g., vaccinations, screenings) but shortages have left 1 in 5 Canadians without a primary care provider. The aim of this study was to compare preventive care uptake between BC rural residents attached and unattached to a primary care clinician. A quantitative cross-sectional survey of rural patients, both with (attached) and without (unattached) a primary care provider, was conducted from July to Sept 2022. Participants completed measures assessing prevention activity completion, priorities, and prevention activity self-efficacy. Descriptive statistics were used to compare preventive care completion and attachment status. A total of 516 rural residents (301 attached; 215 unattached) completed the survey (M age = 50.63 years; 74.4% female). Unattached patients reported lower prevention service completion rates (M = 51%) compared with attached patients (M = 63%; p < .001), although there was no significant difference in the number of prevention priorities. Self-efficacy for provider communication (p < .001), managing chronic illness (p = .002), getting vaccines (p < .001), and completing preventive screening (p < .001) was lower among unattached compared with attached participants. The results indicate a suboptimal uptake of preventive care in rural communities. Furthermore, they highlight a concerning gap in uptake between attached and unattached patients and provide strategic information for developing and implementing preventive care policy and programs, a pressing need given the persistent provider shortage.

The uptake of preventive care services is an essential step toward improving overall health, irrespective of a person’s age, gender, or sex. Preventive care services comprise primary (e.g., immunization) and secondary (e.g., cancer screening) services offered to the general population (asymptomatic) based on age, sex, and risk factors for disease (Krueger et al., 2022). Such services have been shown to avert disease, ensure timely diagnosis and management when disease is detected, and reduce the need for aggressive interventions and associated health care costs (Loftus et al., 2018). Despite the existence of national and provincial guidelines for preventive health care (BC Ministry of Health, 2021b; Canadian Task Force on Preventive Health Care, 2019), overall use has remained low, particularly among residents of rural communities (Loftus et al., 2018; Nelson et al., 2020).

A recent review of U.S. primary care literature found rural-living residents consistently report lower rates of preventive service utilization for various acute and chronic conditions compared with their urban counterparts (Nelson et al., 2020). For instance, the odds of rural patients with a high risk of heart disease receiving primary preventive screening (e.g., lipid testing, glucose testing) were 50% lower than their urban counterparts in the 2 years before acute myocardial infarction; rural seniors were the most affected (Nelson et al., 2020). Unique challenges faced by rural areas in accessing health care include geography, reliable transportation, internet coverage, and financial constraints (Douthit et al., 2015; Maganty et al., 2023). In addition, gaps in access to services in rural communities have long been associated with provider shortages and challenges with retention (Fleming & Sinnot, 2018). Although not specific to rural communities, in 2019, 14.5% of Canadians lacked a primary care provider, with British Columbia (BC) among the provinces with the highest percentage of unattached individuals at 17.7% (Statistics Canada, 2020). Recent survey evidence suggests a worsening trend, with nearly one-third of BC residents reporting they lacked a primary care provider in 2022 (Duong & Vogel, 2023). This may also have been compounded by COVID-19 impacts, with the rate of physicians no longer practicing doubling in the first year of the COVID-19 pandemic compared with previous years (Kiran, 2022).

In Canada, primary care providers are among the first key contacts for receiving many health services, including preventive care. The impact of primary care provider attachment status on preventive care has been examined in previous studies. Compared to attached patients, unattached patients have reported greater self-perceived unmet health needs (Awe et al., 2019), heightened concerns with lack of reminders for preventive care, and less engagement in preventive services (Crooks et al., 2012; Hay et al., 2010). When controlling for demographic and socio-economic status, attached patients were more likely to have better access to care (Kirby & Yabroff, 2020). In their retrospective cohort study using Ontario administrative data, Fitzsimon et al. (2025) found unattachment increased health care utilization and costs, and rose significantly among patients with multi-morbidity. Although they found rural compared with urban location increased health care utilization and costs, they did not disaggregate according to attachment status.

Few studies of preventive care and attachment status consider rurality specifically, despite the greater weight of physician shortages on rural communities given earlier retirements and replacement difficulties, and turnover and retention challenges (Fleming & Sinnot, 2018; Hedden et al., 2017; Silver, 2017). One study on cancer screening found that both rurality and unattachment were associated with lower cancer screening rates (Sabatino et al., 2022). In a survey study of rural patients in a Western Canadian province, the longer patients had been unattached, the lower the proportion of completed prevention activities with only 6.7% of unattached participants up to date on 75% or more of their recommended preventive care activities (Rush et al., 2022). However, primary care provider attachment has been found to be very fluid with many attached patients losing their providers due to retirement or relocation or recently unattached becoming attached (Rush et al., 2025). In addition, evidence has shown that even attached patients may not receive guideline recommended preventive care as providers seek to simultaneously manage chronic disease care, acute care, and documentation (Porter et al., 2023). The current health care landscape, coupled with fluctuating attachment status, may influence preventive care for both attached and unattached patients, but whether this is the case is currently unknown. Therefore, to gain an understanding of preventive care uptake and patient attachment, this study aimed to investigate differences in preventive care uptake between attached and unattached rural patients.

## Method

### Design

**Figure 1:**
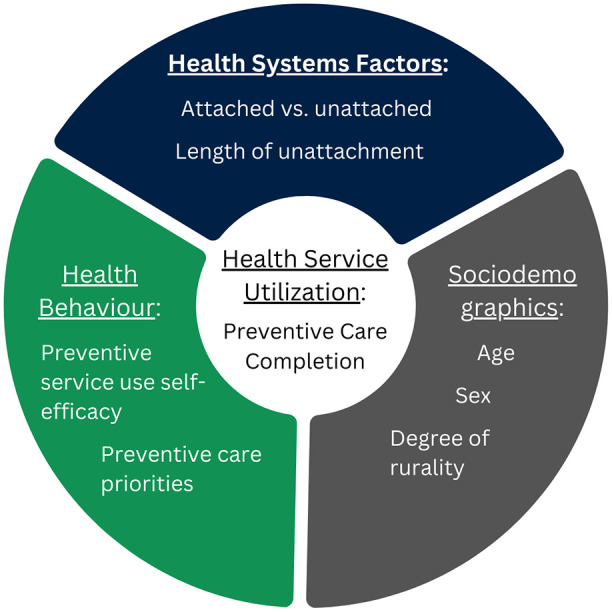
Conceptual Model

A cross-sectional survey design was used to gain a comprehensive understanding of the prevalence of rural preventive care uptake. This study used the core constructs emerging from a scoping review of health care utilization models integrated within the evidence-based BC Lifetime Prevention Schedule Guideline (BC Ministry of Health, 2021a). Core constructs among the models include sociodemographics, health behavior, and health system factors (Gliedt et al., 2023; Figure 1). Variables representing these core constructs were used in data collection and subsequently in analysis to test associations between preventive care completion and priorities and influencing factors. Although health behavior and sociodemographics were examined, this study emphasized health system factors (i.e., primary care provider attachment) in investigating preventive care uptake. The Strengthening the Reporting of Observational Studies in Epidemiology (STROBE) statement (von Elm et al., 2007), guided reporting.

### Sample and Recruitment

Ethics approval was obtained from a university ethics review board (ID# H22-01645). Study participants were recruited between July and September 2022. Individuals above 18 years living in a BC rural community (defined as population < 20,000) were eligible to participate. Rural communities in each BC health authority were identified and targeted. Study advertisements were distributed through rural newsletters, a provincial health research agency (REACH BC), and postings (up to 4) on local community Facebook pages. Targeted ads were utilized to reach under-represented groups (e.g., unattached patients and men). In addition, paid Facebook advertisements were used to augment recruitment efforts in challenging-to-reach areas (i.e., Northern BC) where participation rates remained low.

### Data Collection

Participants completed a 10-min University-hosted online questionnaire, following an online consent form, and could enter a draw for one of three gift certificates valued at CAN $50.00.

#### Social Demographics

Questions asked for age, sex, marital status, ethnicity, level of education, employment, and household income.

#### Geography

Participants selected their home community within a regional health authority and were assigned to a Statistics Canada’s “remoteness index” (RI) group (Alasia et al., 2017; Subedi et al., 2020).

#### Attachment Factors

Questions included whether respondents had a regular primary care provider (yes/no) and length of attachment or unattachment (1–6 months, more than 6 months–2 years, more than 2 years–5 years, more than 5 years–10 years, more than 10 years). Unattached participants were also asked to select reason(s) for unattachment from a predefined list.

#### Chronic Conditions

Participants were asked whether they currently had any chronic illnesses (yes/no).

#### Preventive Activity Completion and Priorities

Seventeen prevention services were selected based on the BC Lifetime Prevention Schedule Guideline and the U.S. Preventive Service Task Force (Rush et al., 2022). These services included cancer screening, screening for asymptomatic diseases or risk factors (e.g., blood pressure), immunizations, and behavioral interventions (e.g., smoking cessation). Respondents self-reported each activity they had completed on a frequency scale—response options included: within the last year, 2 years ago, 3 years ago, 4 to 5 years ago, 6 to 10 years ago, 10+ years ago, or never. Age and sex-specific guidelines were used to assess whether participants were up-to-date with preventive activities (e.g., cervical cancer screening every 3 years for females aged 25–69). For preventive care activities listed in the BC Lifetime Prevention Schedule as “recommended to be routinely offered” (e.g., alcohol and smoking cessation), up-to-date was defined as completed within the past 5 years. Responses were categorized as “up-to-date,” “not up-to-date,” or “not applicable” for each activity. To determine the total proportion of up-to-date scores, the number of prevention activities was divided by the total number of applicable activities for each participant. Participants were also asked to check from a list of 17 prevention services which were priorities for them.

#### Preventive Services Use Self-Efficacy (PRESS)

PRESS is a 16-item validated scale measuring self-efficacy for communication with physicians, self-management of chronic disease, obtaining screening tests, getting vaccinations regularly, and exercise (Jacob et al., 2016). Participants rated their responses on a scale from 1 (*Not at all*) to 10 (*Very confident*). Average scores for each category were calculated based on items in the respective subscales.

### Data Cleaning

Of the 954 survey responses, 516 were retained in analyses. A total of 437 responses were removed due to factors including ineligibility (under age 18 years and/or not living in a rural community; *n* = 7), duplicate participants (*n* = 13), partial surveys (less than half completed; *n* = 62), and suspicious responding (inattentive or potential survey bot responses; *n* = 356). In addition, 25 of the retained participants were missing age. Although this was an eligibility indicator and an important variable when determining relevant prevention activities, it was assumed that participants who did not indicate an age were above 18 years (based on the initial eligibility question) and selected the relevant prevention activities for their age.

### Analysis

Descriptive analyses were conducted using SPSS Version 28. Independent sample *t*-tests were employed to compare the overall proportion of up-to-date prevention activities and preventive self-efficacy scores by attachment status. Chi-square tests were used to examine attachment and whether or not each individual prevention activity was up-to-date. Correlations (Spearman’s rho) were used to examine the association between age, self-efficacy scale scores, and proportion of up-to-date prevention activities. Participants missing age were excluded from age-related analyses. For all analyses, significance was set at *p* < .05 (two-tailed).

## Findings

Participants (*n* = 516) were an average age of 50.63 years (*SD* = 15.0, range 19–86 years), predominately female (74.4%), and White (92.4%). Among the participants, 301 reported being attached to a health care provider, whereas 215 reported being unattached. Mean age was significantly higher for attached (*M* = 52.5, *SD* = 15.0) compared with unattached participants (*M* = 48.0, *SD* = 14.7; Mean difference = −4.49; *p* = .001). Of the remaining socio-demographic variables, only marital status differed between attached and unattached participants (see Table 1); unattached participants were less likely to have been separated/divorced, and more likely to be single, compared with attached patients (*p* = .021).

**Table 1:** Participant Characteristics

Characteristic	Overall, *N* = 516^a^	Attached, *N* = 301^a^	Unattached, *N* = 215^a^	Cramer’s V	*p*-value^b^
**Age group**				.127	.020
18–39	141 (27.5%)	70 (23.3%)	72 (33.5%)		
40–59	175 (33.9%)	100 (33.4%)	75 (34.9%)		
60+	174 (33.7%)	113 (37.5%)	61 (28.4%)		
Missing	25 (4.8%)	18 (6.0%)	7 (3.3%)		
**Sex**				.016	.717
Female	384 (74.4%)	226 (75.1%)	158 (73.5%)		
Male	128 (24.8%)	73 (24.3%)	55 (25.6%)		
Prefer not to answer	4 (0.8%)	2 (0.7%)	2 (0.9%)		
**Ethnicity**				.096	.031
White	465 (90.0%)	279 (92.7%)	186 (86.5%)		
Other Ethnicity	38 (7.4%)	16 (5.3%)	22 (10.2%)		
Missing	13 (2.5%)	6 (2.0%)	7 (3.3%)		
**Marital Status**				.150	.021
Divorced	35 (6.8%)	23 (7.6%)	12 (5.6%)		
Married, Remarried, or Common-law	352 (68.2%)	204 (67.8%)	148 (68.8%)		
Separated	23 (4.5%)	17 (5.6%)	6 (2.8%)		
Single (never married)	80 (15.5%)	37 (12.3%)	43 (20.0%)		
Widowed	26 (5.0%)	20 (6.6%)	6 (2.8%)		
**Education**				.062	.570
Bachelor’s degree	125 (24.4%)	74 (24.6%)	51 (23.7%)		
Diploma or certificate above the bachelor level	97 (18.8%)	53 (17.6%)	44 (20.5%)		
High school diploma or less	120 (23.3%)	66 (21.9%)	54 (25.1%)		
Postsecondary diploma below the bachelor level	173 (33.5%)	107 (35.5%)	66 (30.7%)		
Missing	1 (0.2%)	1 (0.3%)	0		
**Employment Status**				.061	.383
Employed	304 (58.9%)	172 (57.1%)	132 (61.4%)		
Not employed/homemaker/retired	196 (38.0%)	117 (38.9%)	79 (36.7%)		
On disability or medical leave	15 (2.9%)	11 (3.7%)	4 (1.9%)		
Missing	1 (0.2%)	1 (0.3%)	0		
**Income**				.115	.145
Less than $25,000	50 (9.7%)	24 (8.0%)	26 (12.1%)		
$25,000–$49,000	109 (21.1%)	66 (21.9%)	43 (20.0%)		
$50,000–$74,000	104 (20.2%)	62 (20.6%)	42 (19.5%)		
$75,000–$99,000	76 (14.7%)	52 (17.3%)	24 (11.2%)		
Above $100,000	175 (33.9%)	95 (31.6%)	80 (37.2%)		
Missing	2 (0.4%)	2 (0.7%)	0		
**Remoteness Index**					
Easily accessible	0	0	0	.090	.240
Accessible	83 (16.1%)	47 (15.6%)	36 (16.7%)		
Less accessible	143 (27.7%)	91 (30.2%)	52 (24.2%)		
Remote	249 (48.3%)	144 (47.8%)	105 (48.8%)		
Very remote	41 (7.9%)	19 (6.3%)	22 (10.2%)		
**Health Authority**				.090	.376
Fraser Health	8 (1.6%)	6 (2.0%)	2 (0.9%)		
Interior Health	242 (46.9%)	140 (46.5%)	102 (47.4%)		
Northern Health	72 (14.0%)	39 (13.0%)	33 (15.3%)		
Vancouver Coastal Health	52 (10.1%)	36 (12.0%)	16 (7.4%)		
Vancouver Island Health Authority	142 (27.5%)	80 (26.6%)	62 (28.8%)		

*Note*. Participants missing attachment status have been excluded from comparisons.

a n (%). ^b^Chi-square tests (missing responses excluded).

Reasons for unattachment included no doctors accepting patients (*n* = 127, 59.1%), doctor left/retired (*n* = 83, 38.6%), no clinic close by (*n* = 13, 6.0%), provider unavailable after hours (*n* = 11, 5.1%), no clinic found that liked (*n* = 7, 3.3%), no desire to be attached (*n* = 7, 3.3%), and other (*n* = 49, 22.8%). Participant descriptions of “other” reasons for unattachment included those who “*never needed one*,” but also “*a lack of confidence in practitioners*,” often with “*too many doctors rotating in and out*.”

Unattached participants were less likely to have chronic health concerns compared with attached patients (see Table 2). The total proportion of up-to-date personal prevention activities ranged from 0% to 100% among both attached and unattached participants. Overall, unattached patients reported lower prevention service completion rates (*M* = 51%) compared with attached patients (*M* = 63%), although there was no difference in number of prevention priorities. The longer participants had been unattached, the lower the proportion of completed prevention activities (Spearman’s *r* = −.39, *p* < .001), but unattachment time was unrelated to number of prevention priorities (Spearman’s *r* = −.13, *p* = .061).

**Table 2:** Health Characteristics and Preventive Activity

Characteristic	Overall, *N* = 516^a^	Attached, *N* = 301^a^	Unattached, *N* = 215^a^	Cramer’s *V* / Cohen’s *d*	*p*-value^c^
**Presence of Chronic Health Concerns**				.182	<.001
No	219 (42.4%)	105 (34.9%)	114 (53.0%)		
Yes	295 (57.2%)	194 (64.5%)	101 (47.0%)		
Missing	2 (0.4%)	2 (0.7%)	0		
**Prevention Activity Up to Date (%)**	57.97 (22.89)	63.15 (20.49)	50.73 (24.13)	−.562	<.001
**Prevention Up-to-date Quartiles**				.263	<.001
0–24%	61 (11.8%)	21 (7.0%)	40 (18.6%)		
25–49%	128 (24.8%)	59 (19.6%)	69 (32.1%)		
50–74%	191 (37.0%)	123 (40.9%)	68 (31.6%)		
75–100%	136 (26.4%)	98 (32.6%)	38 (17.7%)		
**Number of Prevention Priorities**	5.81 (3.6)	5.86 (3.72)	5.74 (3.66)	−.032	.722
Missing	1	0	1		
**Exercise *SE***	7.84 (2.45)	7.85 (2.46)	7.84 (2.45)	−.005	.954
Missing	10	6	4		
**HCP Communication *SE***	6.38 (3.15)	7.48 (2.59)	4.71 (3.20)	−.973	<.001
Missing	30	8	22		
**Disease Management *SE***	7.37 (2.50)	7.69 (2.21)	6.93 (2.80)	−.306	.002
Missing	52	30	22		
***SE* for Getting Vaccinated**	7.41 (3.06)	7.96 (2.86)	6.63 (3.19)	−.443	<.001
Missing	64	36	28		
***SE* for Completing Preventative Screenings**	6.22 (2.93)	7.01 (2.67)	5.13 (2.92)	−.675	<.001
Missing	25	16	9		

*Note. SE*, self-efficacy; HCP, health care provider. Participants missing attachment status have been excluded from comparisons.

a Mean (*SD*); *n* (%). ^c^Chi-square tests; Independent-samples t-tests (missing responses excluded).

For attached patients, the length of time they had been with a health care provider was unrelated to the proportion of completed prevention activities (Spearman’s *r* = −.07, *p* = .244), nor to number of priorities (Spearman’s *r* = .01, *p* = .869). Remoteness was unrelated to the proportion of services participants were up to date on overall (Pearson’s *r* = −.05, *p* = .304). There was no difference between attached and unattached participants in self-efficacy for exercise; however, self-efficacy was lower among unattached participants for health care provider communication, managing chronic illness, getting vaccines, and completing preventive screening compared with attached participants.

Completion rates of individual prevention activities ranged from a low of 18% (*hearing test*) to a high of 90% (Chest CT; see Table 3). In terms of individual prevention activities, compared with attached, unattached participants were less up to date on cervical cancer screening, mammogram, eye pressure testing, dental cleaning, cholesterol screening, blood glucose check, blood pressure check, influenza vaccine, tobacco cessation, mental health check-in, and alcohol screening.

**Table 3:** Proportion of Attached and Unattached up to Date on Each of the 17 Prevention Activities

Activity	Recommended population and schedule	Not applicable	Overall *N* = 516^a^	Attached *N* = 301^a^	Unattached, *N* = 215^a^	*p*-value^c^
Cancer Screening						
Cervical Cancer Screening	Females age 25–69 every 3 years	[179]	224 (66.5%)	140 (73.3%)	84 (57.5%)	.002
Colonoscopy/FOBT	Adults age 50–74 FOBT every 2 years OR colonoscopy every 10 years	[255]	179 (68.6%)	121 (72.0%)	58 (62.4%)	.107
Mammogram	Females age 50-74 every 2 years	[331]	121 (65.4%)	87 (73.1%)	34 (51.5%)	.003
Chest CT Scan	Individually requisitioned, so relied on participant reports	[350]	149 (89.8%)	101 (92.7%)	48 (84.2%)	.088
Screening for other asymptomatic diseases and risk factors						
Eye Pressure Test	Adults age 19+ at least every 2 years	[0]	293 (56.8%)	185 (61.5%)	108 (50.2%)	.011
Dental Cleaning	Adults age 19+ at least once a year	[0]	314 (60.9%)	202 (67.1%)	112 (52.1%)	< .001
Cholesterol Screening	Males age 40+ and females age 50+ every 1–5 years	[221]	220 (74.6%)	154 (81.1%)	66 (62.9%)	< .001
Blood Glucose Check	Adults age 40+ every 3 years	[150]	230 (62.8%)	154 (68.4%%)	76 (53.9%)	.005
Blood Pressure Check	Adults age 18+ at every appropriate visit	[0]	297 (57.6%)	197 (65.4%)	100 (46.5%)	< .001
Hearing Test	Adults age 60+ every 2 years	[335]	33 (18.2%)	20 (17.1%)	13 (20.3%)	.592
Bone Density Testing	Males age 70+ and females age 65+ at least once	[411]	56 (53.3%)	41 (54.7%)	15 (50.0%)	.665
Immunizations						
Tetanus Vaccine	Adults age 19+ every 10 years	[0]	112 (54.5%)	167 (55.5%)	114 (53.0%)	.580
Influenza Vaccine	Adults age 19+ yearly	[0]	257 (49.8%)	166 (55.1%)	91 (42.3%)	.004
Pneumococcal Vaccine	Adults age 65+ once	[404]	45 (40.2%)	32 (41.6%)	13 (37.1%)	.659
Behavioral Interventions						
Tobacco Cessation	Adults age 19+ “routinely offered”	[90]	355 (83.3%)	221 (87.7%)	134 (77.0%)	.004
Mental Health Check In	Adults age 19+ “routinely offered”	[0]	191 (37.0%)	126 (41.9%)	65 (30.2%)	.007
Alcohol Screening	Adults age 19+ “routinely offered”	[0]	289 (56.0%)	182 (60.5%)	107 (49.8%)	.016

*Note*. Participants missing attachment status have been excluded from comparisons.

a n (%). ^c^Chi-square tests (‘Not applicable’ responses excluded).

The proportion of attached compared with unattached participants who prioritized each relevant prevention service is shown in Table 4. Only prioritizing hearing test was significantly different with more attached participants prioritizing the test (45.3%), than unattached participants (26.5%). Overall, dental cleaning (68.2%), cervical cancer screening (62.0%), mammogram (62.2%), colonoscopy (49.4%), blood glucose (49.5%) and blood pressure check (55.6%) were the highest prioritized activities, whereas tobacco cessation (3.8%) and alcohol screening (8.9%) were the lowest.

**Table 4:** Proportion of Attached Compared to Unattached Participants Who Selected Each Prevention Service as a Priority (of Those for Whom Each Activity Was Relevant)

Activity	Attached		Unattached		
	Eligible *N*	Yes, selected as a priority *N* (%)	Eligible *N*	Yes, selected as a priority *N* (%)	*p*-value
Cancer Screening					
Cervical Cancer Screening	191	113 (59.2%)	146	96 (65.8%)	.217
Colonoscopy/FOBT	93	48 (51.6%)	168	81 (48.2%)	.599
Mammogram	66	41 (62.1%)	119	74 (62.2%)	.993
Chest CT Scan	57	26 (45.6%)	109	33 (30.3%)	.050
Screening for other asymptomatic diseases and risk factors					
Eye Pressure Test	301	137 (45.5%)	215	91 (42.3%)	.472
Dental Cleaning	301	202 (67.1%)	215	150 (70.0%)	.523
Cholesterol Screening	105	33 (31.4%)	190	64 (33.7%)	.693
Blood Glucose Check	141	70 (49.6%)	225	111 (49.3%)	.954
Blood Pressure Check	301	171 (56.6%)	215	116 (54.0%)	.520
Hearing Test	64	29 (45.3%)	117	31 (26.5%)	.010
Bone Density Testing	30	12 (40.0%)	75	29 (38.7%)	.899
Immunizations					
Tetanus Vaccine	301	63 (20.9%)	215	51 (23.7%)	.451
Influenza Vaccine	301	114 (37.9%)	215	69 (32.1%)	.176
Pneumococcal Vaccine	35	11 (31.4%)	77	26 (33.8%)	.807
Behavioral Interventions					
Tobacco Cessation	174	7 (4.0%)	252	9 (3.6%)	.810
Mental Health Check In	301	135 (44.9%)	215	85 (39.5%)	.229
Alcohol Screening	301	26 (8.6%)	215	20 (9.3%)	.794

*Note*. CT, computed tomography; FOBT, fecal occult blood testing.

## Discussion

This is the first study to compare preventive care uptake between attached and unattached rural patients and find overall low uptake across patient groups (58%) but lower uptake with unattachment (51% vs. 63%). Further 7% of attached and 20% of unattached patients had less than 25% of preventive activities up to date. Length of unattachment was related to fewer preventive care services completed. These findings are consistent with an older systematic review that showed having a usual source of care was associated with improved receipt of preventive services (Kim et al., 2012). A U.S. study found that having a usual place and a usual provider was consistently associated with increased odds of receiving preventive care services (Blewett et al., 2008). Similarly, a Canadian study found unattached patients were three times less likely to report receiving routine care such as monitoring of health issues or check-ups compared with attached patients (26% vs. 73%; Hay et al., 2010).

Between group preventive services completion rates differed depending on the need for physician referral. The uptake of individual services that required physician referrals (e.g., mammography, colonoscopy) and those not requiring a physician referral (e.g., flu immunization) were significantly lower for unattached compared with attached patients. This discrepancy in the non-referral services may be attributed to physician encouragement of their patients to complete preventive care activities during episodic care. In a study of provider approaches to preventive care in the United States, providers reported that an established relationship with their patients was integral to preventive care and were hesitant to provide such care with unattached patients (e.g., walk-ins; Murugan et al., 2018). Furthermore, family physicians’ lack of time and heavy workloads limit their ability to provide health promotion and prevention services (Luquis & Paz, 2015). Preventive services accessed through other health professionals (e.g., dentist, optometrist, pharmacy), saw similarly lower completion rates among unattached patients. This perhaps reflects similar shortages of health professionals in rural locations.

Except for exercise, self-efficacy for health care communication, managing disease, screening, and immunizations, was lower among unattached participants. This finding might reflect a sense of defeat, given that unattached patients prioritized preventive activities similar to attached patients but faced barriers to accessing care. Dental was the most prioritized activity, followed by several screening activities with alcohol use and tobacco check-ins the least prioritized, potentially reflecting stigma or a lower proportion of participants using these substances. Notably, blood pressure check was prioritized by over half of both attached and unattached participants and was among the most cost-effective preventive services in a BC analysis (Krueger et al., 2022).

In the present study, rural unattached patients were more up-to-date (50.5%) compared with a 2020 survey of rural unattached patients, where the average prevention completion rate was 39.6% (Rush et al., 2022). However, COVID-19 may have played a role in restricting access to services due to the shutdown of services in the early waves of the pandemic. In a Canadian study, Laing and Johnston (2021) found preventive care screening rates had decreased for cervical cancer screening, colorectal cancer screening, and Type 2 diabetes screening in the 38 weeks since March 15, 2020.

### Strengths/Limitations

This study makes a novel contribution in addressing a gap in the literature related to rural attachment status and its impact on unattached patients. It is the first study to show that despite both attached and unattached patients prioritizing preventive services similarly, completion of many preventive activities, not just those requiring physician referral, were lower for unattached patients. However, there were some limitations. Focus on rural communities in BC limits finding generalizability to urban or suburban contexts and other regions. Although there were no participants from “easily accessible” communities, some participant communities were considered “accessible” due to their proximity to larger centers, despite still being considered rural, with populations well below 20,000, and many with Canadian rural regional postal codes (Canada Post, 2022). There was representation from each health care region across various rural and very remote locations. However, the online survey methodology may have excluded residents with inadequate internet access or broadband connectivity.

Despite targeted recruitment efforts, 74.4% of the sample was female, reflecting the ongoing challenge of recruiting males (Rush et al., 2022). In addition, the sample was predominantly White, with 92% identifying as such, limiting generalizability, particularly concerning diverse ethnic groups, such as Indigenous populations. Research on preventive care in Indigenous communities, especially those in rural and remote areas, is limited (Ireland et al., 2023). Future studies should focus on ethnically diverse groups to better understand the impact of provider attachment and various systemic barriers affecting the utilization of preventive care services.

Participants’ self-reported prevention service uptake may have introduced bias due to overlooking or misremembering details about the services and their timing or considering services as relevant despite not being pertinent. While this study explored participants’ preventive care priorities, a more nuanced analysis is required to determine the interplay between prevention completion and priorities along with other variables. For example, that unattached patients were younger, less likely to have chronic conditions, and more likely to be single may have played a role in other differences between these two groups, such as prevention completion. We used a conceptual model descriptively to categorize variables in this study (Gliedt et al., 2023), but future work might seek to examine the model’s predictive validity.

### Implications for Practice

Findings from this study have implications for practice to benefit preventive service use for both attached and unattached patients. Health educators, with unique expertise in behavior change, health promotion, and disease prevention, could play an important role in preventive services use (Bruening et al., 2018). They could boost unattached peoples’ self-efficacy, lower in the current study, through goal setting, problem-solving, and targeted education (Acharya et al., 2021). Often positioned across a range of health care settings (e.g., health departments, community organizations, businesses, hospitals), they may be more accessible to those without a primary care provider. For example, they may be more accessible to unattached younger participants, who may benefit most from targeted digital approaches (Blue Cross, 2024). Including health educators in team-based primary care models has increased preventive service uptake compared with solo physician models (Schor et al., 2019) and leverages their expertise for attached patients.

Unattached participants’ lower self-efficacy, possibly driven by lower health literacy or knowledge, might be addressed through health communication campaigns, peer mentoring, or community screening events. These events could also address the disparity in blood pressure, cholesterol screening, and blood glucose screening. Further personal health records or self-screening, such as self-screening cervix kits (BC Cancer, 2024), could also increase self-efficacy and preventive care uptake. Automated telephone communication systems, used successfully to prompt and remind about screening and immunizations (Posadzki et al., 2016), might be used to reach both attached and unattached patients, potentially alleviating the burden from primary providers who are squeezed to provide episodic services with the time needed to treat not considered in guideline recommendations (Johansson et al., 2023; Porter et al., 2023).

Although prevention priorities were similar among both attached and unattached patients, focusing provider attention on only high-priority preventive services might enhance their capacity to integrate prevention into the care of patients not part of their caseload. This might occur either through patient self-reported rankings, as in the current study, or through an individualized decision-making algorithm that uses patient-specific clinical characteristics for prioritizing (Taksler et al., 2013).

### Implications for Policy

Policy makers need to address the primary provider attachment situation, which has left many rural patients in crisis over their health care management (Rush et al., 2025). Although efforts have been made, it is imperative that policy makers expedite the attachment process as the longer patients are unattached the worse their preventive care uptake becomes. In the meantime, government policy needs to include guidelines to support unattached patients in meeting their health care needs during periods of unattachment, particularly those from ethnic populations who have among the highest unattachment rates (Bayoumi et al., 2023). Importantly, missing is a mechanism for ensuring adequate follow-up, particularly when results are abnormal. Similarly, addressing cost barriers, such as expanding dental and optometry services coverage in BC, could improve completion rates.

## Conclusion

The findings indicate a significant shortfall in preventive care uptake within rural communities. Moreover, they underscore a troubling disparity in uptake rates between individuals with an established primary care provider and those without, emphasizing the imperative for targeted assistance for the latter group. Given the anticipated ongoing shortages of health care providers, proactive solutions to enhance preventive care engagement are imperative.
